# Supplementary material for: Informing State-Wide Coalition Efforts to Implement and Integrate Nutrition Best Practices in Early Care and Education: Focus Group Insights from Child Care Providers
Source: Int J Environ Res Public Health. 2022 Aug 14;19(16):10025. doi: 10.3390/ijerph191610025 (PMC9408123; doi:10.3390/ijerph191610025)
Supplement: Supplementary file 1 [file ijerph-19-10025-s001.zip › ijerph-1820799-supplementary.pdf]

Table S1. Themes, descriptors, and illustrative quotes from focus group participants

Code/theme	Description	Illustrative quote
**Reasons providers became interested in supporting children's health**		
**Personal health motivation**	Providers are motivated by their own health struggles including cancer, obesity, and age, as well as their own health successes (e.g., losing weight after following CACFP guidelines).	"Well, the reason that we have adapted toward more organic and unprocessed foods and canned foods. I had thyroid cancer and when we were going through certain parts of the diet plan like I could not absolutely have anything with like canned food, it was not allowed because of some of the contents that could have been inside of it, so after that it was just we starting off fresh vegetables, so anything from fresh vegetables to the more organic or less processed food to keep our health in check and understand. So then we know our body and what is making it different." (Participant 5.7)
**Love and passion for kids and their health**	Providers are aware of the obesity epidemic and want to learn more about children's health.	"Oh, what motivates me to do that is. I mean just to see the kids. I mean, make sure that they're growing right and you know, just keeping them healthy. I mean like, I mean because me I'm a health freak, I mean gym is my big thing, so we dance all the time. We're always moving, I mean just to see them you know, be healthy." (Participant 1.3)
**Licensing and beyond**	Licensing standards were motivating in the beginning, but over time experience has become more of a motivating force.	"It well it goes with that [licensing standards]. That's part of it, but then it goes beyond it. Just gotta have a passion for what you doing for what you want to do. Right now. I chose this path. You know I chose this profession to work with children. So each day I get up, I'm motivated and ready to do different and challenging things with them. So that motivates me every and all day, yeah, and it's like you only say being a mother, a mother of six in a grandmother 15 and then I have a daycare children. And then I'm in school getting my Masters, so I'm really I'm really motivated." (Participant 3.4)

**Ways providers currently support children's health**		
**Ensuring cleanliness**	Providers clean and sanitize toys, surfaces, wash hands, remove shoes and practice food safety.	"Um, as far as when they arrive, wash your hands and move their shoes and socks and jackets, hand sanitizer their hands, wipe down the area where they're gonna be doing that E-learning working it and go from there." (Participant 3.4)
**Promoting physical activity**	Providers keep kids active and outside doing a variety of physical activities.	"We're outside almost all the time except for the 20 minutes we're inside, doing circle time and craft. If it's an indoor craft and in the summer is when we're outside, I'll be honest. We don't wear shoes or anything like that, so. We're getting you know, the natural environment. It's great." (Participant 5.4)
**Focusing on healthy eating**	Providers serve organic foods, no processed foods, correct portions, and balanced meals	"For me, I cook all the meals for daycare, so I just make sure that they're getting balanced meals and that they're getting the right serving sizes and then just pushing a lot of vegetables, fruits and everything else, just making sure that they're eating balanced meals" (Participant 1.5)
**Modeling and educating about healthy habits**	Providers model healthy eating and personal hygiene. Providers educate parents and children about healthy eating, hygiene, and medical care.	"We sit down as teachers and model that same behavior ["take a polite bite"], you know we're eating the vegetables. We're eating whatever the kids don't like. Let them see what we do and try to ask them to take a 'polite bite' of the things they don't like." (Participant 2.2)
**Ways providers implement nutrition best practices**		
**Using information/resources and following guidance of nutrition programs like CACFP**	Providers use resources from CACFP like nutritionists, newsletters. They do not allow children to bring food from home. They attend other nutrition related trainings.	"The nutrition program [CACFP], and the newsletter and the training they have there. That has really helped me to be able to implement, you know, have my thought, my thinking about nutrition." (Participant 1.2)
**Heathy substitutions or preparations**	Providers roast instead of fry foods, serve fruit as dessert, avoid juice and canned foods, and mix whole grain and regular pasta together.	"Well I could say at first I had a challenge with the whole grain pasta. So I mixed it together so they wouldn't be able to tell the difference. You know so I just added the regular pasta that they were used to eating and now it's ok." (Participant 1.3)

**Including children and parents in meal planning, prep and leaning about healthy eating**	Providers talk about healthy eating habits, share recipes with parents, and ask for input and participation from kids and parents.	“One of the projects that I began doing with the children last year was a garden. They grew their own vegetables, the ones that they were interested in. They took care of them and at the time of harvest, they gladly accepted them because it was something they were invested in.” (Participant 4.2)
**Challenges to implementing nutrition best practices**		
**Picking eating**	Kids resist vegetables. They do not like to try new foods. Kids want processed or fast food.	“You know the kids, if they’re not used to eating something. Sometimes they look at it and say ‘I don’t wanna taste. I don’t wanna eat this.’ So it kind of be challenging just to, you know, get them to taste it.” (Participant 1.3)
**Negative influence of parents**	Parents set poor examples at home (too much processed/fast food). They want to send it with kids to day care.	“Seeing what these kids come into the daycare center from home…what they bring in, hot Cheetos for breakfast. Instantly, you know, like to ‘Let me switch this out for a granola bar or a cereal bar’ just to give them something healthy and say ‘It’s OK to eat this every now and then,’ but watching this kid coming every day with McDonald’s, hot Cheetos, Hi-C juices, you know, like ‘OK, we have to do something about it.’” (Participant 2.2)
**High cost of food**	Food is expensive, especially healthy, fresh food.	“I think it just goes back again to the prices in the expense, because in the summer you can get such a big variety or fruits and vegetables, there's no issues, but once it starts getting cold kind of start running into limits of what you can get, that's not going to cost an arm and a leg. So in the summer time is fantastic. I can buy anything for cheap and the kids get beautiful selections as it gets to winter I kinda have to, you know, budget a little bit more and pay attention to it a little bit more. And I'm not gonna spend a ton of money and things that I'm not sure they're like, so I'll be a little bit less willing to try things with the kids in the winter when it's more expensive.” (Participant 1.5)
**Access to healthy food**	Groceries stores are far away. It is hard for providers to find what they need. It is hard to find foods that meet requirements for sugar.	“Everything has risen and also in certain areas that we stay in, like you can't go to a specific grocery store. They might not have, they might have to go 25 miles out to get it” (Participant 3.7)

**Ways providers approach caring for infants who are breastfed or given expressed milk**		
**Provides space and physical supports for mothers**	Parents have a private space and supplies to feed or pump and are welcome at any time.	"We encourage it. If they're, if they're close, we have a section in our classroom, that if a mom wants privacy to nurse, she's more than welcome to go back there. If she wants to hang out with us and nurse, that's totally fine too. You know, we provide boppies anything like that kind of aid with holding and all that." (Participant 5.6)
**Provides emotional support, encouragement and information to mothers**	Provider is a resource and source of encouragement to feeding parents. Provider follows wishes of parents and serves as a support.	"To be very patient. Very patient, very understanding. Like she said early, I breastfed too so. But to really work with them so they don't get discouraged." (Participant 1.2)
**Gives extra attention to breastfeeding and transitioning babies**	Nursing or expressed milk fed babies are given extra attention, especially when they are transitioning to bottle or formula.	"Sometimes we just take more time to just love on that baby because it transition can be hard. So it gives us more opportunity to have one-on-one with that child." (Participant 2.3)
**Breastfeeding knowledge, training, and outside support**		
**Additional training desired (for provider and parents)**	Providers want to learn more about cultural norms, creating a baby friendly feeding environments, mechanics of breastfeeding, and how to convey benefits of breastfeeding to parents.	"I think training it would be nice because I do like where it says develop plan for introducing age appropriate foods. I usually like the other provider said I typically waited for the parents to tell me or if I was curious about it, I would ask, but it would be really cool to have a plan and have like even forms ready that you talk to the parents. You sit down and say Hey, this is what I think it would just make us be a little bit more confident that will make us look a little bit more professional instead of waiting for the parent to bring it up. So a little bit extra training on this would be nice." (Participant 1.5)
**No additional training desired**	Some providers feel they do not need any additional training.	"At our center we do all these things. So we support it completely." (Participant 2.3)
**Additional desired support from other sources**	Providers wish physicians, the community, family, and friends would all provide more support for breastfeeding families.	"Think definitely their physicians, primary care physicians. Community support. I feel like it should be normalized. It's 2020, but yeah I feel like you know friends, family, primary care physician for sure." (Participant 5.6)

**Provider has no formal training around breastfeeding**	Providers' knowledge around breastfeeding comes from personal experience or from family and friends.	"My kids. Me. Myself. My daycare kids and then when it was time for my daughters then I could throw on some information to them. But then they threw information back to me and then I change that to adding it to daycare now. You know 36 years you learn a lot [about breastfeeding]." (Participant 5.2)
**Implementing and supporting screen time best practices**		
**Virtual learning requires excess screen time**	Providers are concerned about how much screen time is required for school-aged children.	"I am concerned about the situation of virtual classes that school-age children are currently having because now they hardly get to go out. They do not get to accompany us to play outside when we go outside to play. They spend all… practically all day in front of their tablet or their computer listening to their classes. And now that the cold weather is coming, they will get to go out much less. So, there will be a little bit of a problem with implementing physical activity because even though they have virtual PE classes, it is not the same for them to exercise in front of a screen. We shall see what comes out of all this." (Participant 4.4)
**Parents allow excess screen time**	Providers are concerned that parents send tablets with kids and have few technology restrictions at home.	"That's always an issue [sleepy children arriving in the morning]. Keeping her up all night watching TV and so by the time they reach daycare, they're too sleepy to even [eat] breakfast." (Participant 3.3)
**Desire for more support around screen time best practices**	Providers want more information about screen time best practices. They know it is not good but do not always know why.	He's like obsessed with Sesame Street…I'm thinking like, well at least he's learning. I can tell he's not even two, and he knows so much for watching this stuff, and I feel like you know. I'm torn between should I allow him to it as much or take it from him?" (Participant 1.4)
**Provider already implements best practices around screen time**	Some providers feel they are already implementing best practices for screen time.	"The majority of it, is things I already do" (Participant 3.3)
**Active screen time**	Some providers allow some screen time. Sometimes as a means of exercise, learning, or reward for other	"Here at our daycare center, we're only allowed maybe 10 minutes and preschool of screen time. Whether it's computer, showing the kids a short video, what we're incorporating that week, we're only allowed

	completing tasks or following directions.	so much. The screen time here, because you never know what kids are getting at home.” (Participant 2.2)
**Passive screen time**	Some providers leave screens on in the background during drop-off or pick-up, or during meals.	“For me, screen time is, in the morning when I open at six 6:30, I turn the TV on. 7:30 TV goes off and it's all for the rest of the day. It may come back on as time for the children to go home at 5/5:30, and by the time it comes back then, most of them are exiting out so.” (Participant 3.4)
**Needed support and sources of support**		
**Desire for more training and support overall**	Providers want more training and support around meal and snack ideas, expanding the variety of foods they serve, how to better engage parents. They also want additional funds and updated technology.	“We should have more healthy nutritional trainings. We had them in the past because I enjoyed the cooking and eating, but more healthy nutritional trainings. You know more trainings for parental help. Parents can help outside of just dropping your child and not doing anything else” (Participant 3.4)
**No additional support needed**	Some providers do not want additional support.	“Because you used to it, you know in my case, I'm just used to, I know what to give them what they can't have. No challenge.” (Participant 1.4)
**Trusted sources of information**	Providers trust many sources of information, including CACFP, child care provider networks, INCCRRA, UIUC extension, YWCA, and other related organizations.	“The healthy food program. So the Food Program is one that usually they sent fliers every month and they give us ideas. So that's been one and I'm in Chicago, some with Action for Children, so I follow them and I get ideas from them that somebody that I would trust.” (Participant 1.5)
**Where providers go for information**	Providers have access to many sources of information including, CACFP, conventions and conferences, other courses, and trainings, We Choose Health Texting Club, Just Move program, google, INCCRRA, and medical professionals.	“Sometimes through my director or I do read up on INCCRRA a lot, too. Gateways, like they have a lot of information.” (Participant 3.2)
